# Psychiatrists’ awareness of the cost of medication, rates of social disability pension, and their attitudes regarding knowledge of treatment costs

**DOI:** 10.1186/s13584-022-00545-6

**Published:** 2022-10-10

**Authors:** Ido Lurie, Saed Maree, Shlomo Mendlovic, Daphna Shefet

**Affiliations:** 1 Shalvata Mental Health Center, P.O.B 94, Hod Hasharon, Israel; 2 Sackler School of Medicine, Tel Aviv University, Tel Aviv, Israel

**Keywords:** Attitudes, Economic aspects of mental health care, Psychiatrists’ awareness, Psychotropic medication costs

## Abstract

**Background:**

Medication is a significant component of the cost of mental health care. Studies from different countries indicate that physicians are often not aware of medication costs, despite the impact of such knowledge on treatment plans. The purpose of this study was to examine Israeli psychiatrists’ knowledge regarding the cost of medication and rates of disability pension, and the impact of these factors on treatment decisions.

**Methods:**

Cross-sectional study. A questionnaire was distributed by e-mail to psychiatry specialists and residents, including: (a) socio-demographic and professional information; (b) knowledge regarding rates of National Insurance disability pension and medications’ costs; (c) attitudes regarding relevance of knowledge of treatment costs. Correlations between socio-demographic variables and knowledge regarding treatment costs and attitudes were examined.

**Results:**

Of the 175 psychiatrists who completed the questionnaire, 55% were men. The level of knowledge regarding cost of psychotropic medication and disability pension rates was low. Sixty-eight percent rated economic considerations as important or very important, yet 75% were informed of medication prices by their patients, and 57% by pharmaceutical companies. Doctors who worked in regions other than the center of the state were more aware of the economic aspects of treatment. Physicians who reported easy access to information regarding drug prices were less likely to err in estimating the price of medications.

**Conclusions:**

Psychiatrists in Israel are not sufficiently informed of the costs of psychotropic medications, despite their awareness of the relevance of affordability to treatment compliance. Awareness of economic issues relating to treatment should be included in residency programs, and access to relevant information of medication cost and disability pensions should be more accessible to physicians both on national and local levels, by the government and health maintenance ogranizations (HMOs).

## Background

Mental illness has associated direct and indirect financial implications. Among other considerations, cost of mental health care, whether private or public, impacts the choice of psychotropic drug treatment. This is especially important as many patients, particularly those with severe mental illness, have limited economic resources (e.g., National Insurance Institute disability pension) and are often unable to keep a job. In Israel, the employment rate among persons with mental disabilities is only 46%, and among those with dual disabilities, physical and mental, employment decreases to 38% [1].

There are often significant discrepancies in the cost of psychotropic medications with similar indications and efficacies. Awareness of the cost of medical treatment in general and pharmacological treatment in particular, as well as the medical responsibility for utilizing this information is not currently included in the medical curriculum or training for various cultural, social, and historical reasons [2]. Studies conducted in different countries indicated low awareness of physicians to the importance of both knowledge and consideration of cost when prescribing medications [3–5].

Surveys of physicians regarding economic issues associated with psychiatric treatment have been conducted in numerous countries. A study conducted in Norway [4] examined the knowledge of general practitioners (GPs) regarding the cost of five common drugs, including the anti-depressant escitalopram, found that 53% of physicians would price it at less than half its actual cost. A survey of physicians in the United States (US) indicated that only 20% were aware of patients’ out-of-pocket expenses when prescribing medications [3]. A systematic review of 24 studies on this topic found that in only 31% of cases the cost estimate was in the range of 20–25% of the actual price, with a tendency to overestimate the cost of inexpensive medications, and to underestimate the cost of expensive medications [5].

An Israeli study of one hundred GPs found that only a minority correctly estimated the cost of common tests such as chest x-rays and blood counts [6]. In that study, 78% of the physicians reported that they were never educated in the field, and an equal percentage was interested in acquiring such information. Interventional research among over 150 physicians in the US included the distribution of a drug price booklet and a lecture. After six months, 54% reported using the booklet at least once a month, and two-thirds rated its effectiveness as moderate or high [7].

A survey among psychiatrists (N = 265) in the US regarding awareness of mental health care costs, including pharmacotherapy, psychotherapy and laboratory and imaging tests, showed that psychiatrists thought that this knowledge is essential to the process of choosing drug treatment, and that they were confident in their knowledge regarding the costs. However, the level of knowledge regarding drug costs was uneven [8]. Most of the estimates of medication prices were inaccurate (the accuracy ranged between 0 and 41%), with an underestimation mainly of original name brand drugs (Fluoxetine- Prozac, Valproic Acid- Depakote). However, 42% reported that they usually considered costs when making therapeutic decisions and 50% reported that they felt informed or very aware of treatment costs. Over 39% answered that consideration of costs when making a treatment decision is “very important” or “extremely important.” The majority (61%) answered they could provide better service to their patients with more information about treatment costs.

A UK survey was conducted among psychiatrists who worked for the North East London Foundation Trust between November 2007 and January 2008. The survey was completed by 71% (102 out of 143) of doctors working in adult or old age psychiatry. Only 2% of the doctors stated that they should always take into consideration the price of the drug when prescribing and only 5% claimed to know the price of medications that they prescribe most frequently. Of these, 17% could estimate the prices within 20% above/below the actual cost. A total of 35% of the doctors stated that they had, within their hospitals, ready access to the cost of the drugs they prescribe [9]

Medical insurance in Israel is mandatory, thus healthcare is available for citizens and residents as a fundamental right. According to the National Health Insurance Law of 1994 [10] all citizens and residents are required to be enrolled in one of four official health maintenance organizations (HMO), which are non-profit organizations and are prohibited by law from denying any Israeli resident membership. Mental health services, including some but not all psychotropic medications, are included in HMO insurance plans since 2015.

The aims of this study were twofold: (1) to assess the knowledge of psychiatrists, both specialists and residents, regarding the costs of psychotropic pharmacotherapy and the financial status of patients with psychiatric diagnoses (i.e., National Insurance Institute disability pension, and the percentage of HMO participation in the purchase of medications); (2) to examine psychiatrists’ attitudes regarding the importance of awareness of these issues when making treatment decisions.

To date, no such survey has been conducted in Israel, despite its relevance to patients, their families and caregivers, and on the broader level, healthcare economics.

## Methods

### Study sample

Between November 25, 2018 and January 29, 2019, the study’s survey questionnaire, based on Qualtrics—an online questionnaire platform, was distributed via email to the list of psychiatric specialists in the Israeli Medical Association (IMA). Out of approximately 1100 psychiatrists, 275 doctors responded, and of those, 171 completed the entire questionnaire. As only specialists in psychiatry are listed, resident psychiatrists were contacted using a separate list, with snowball recruitment. Of those, 24 residents completed the entire questionnaire. A letter of approval and encouragement to participate from the chairperson of the Israeli Psychiatric Association (IPA) was distributed together with the questionnaire. The study was approved by the Shalvata Mental Health Center institutional review board (IRB). No identifying details of study participants were collected in the survey. Completion and submission of the questionnaire was considered consent to participate in the study.

### Survey instrument

The anonymous questionnaire included three sections:

(a) Socio-demographic and professional information, including: age, gender, marital status, country of birth, country of medical education and psychiatric residency, stage of residency, work place location- center or other, adult or children and adolescent specialization.

(b) Knowledge questions regarding: 1. the amount of National Insurance Institute disability pension; 2. the cost of specific medications, including anti-depressants, anxiolytics, anti-psychotics, attention deficit hyperactivity disorder (ADHD) medications, medicinal cannabis; 3. rates of patients’ deductibles by the HMOs (HMO maximum coverage).

(c) Questions regarding attitudes towards awareness of medication costs, including: Do you think it is important to know the cost of medication? Is the cost of a specific medication taken into account when choosing a treatment plan for your patients? Have you ever asked a patient about his/her ability to purchase the medication before prescribing? What is the source of your knowledge regarding medication costs? (patients, pharmacies, pharmaceutical companies’ websites, brochures, salespersons, HMO updates, other); How easy is it to acquire information about the cost of psychotropic medications?

### Data analysis

After collecting data and prior to analyses, data cleansing was performed, by identifying and handling outliers and missing values. The study examined two main groups of dependent variables. First, gaps between subjective estimations of psychiatric medication costs and actual objective costs (derived from the Ministry of Health price list)^1^ were calculated. Final gaps represent the deviation by percentage from actual objective costs. Positive deviations mean that psychiatrists overestimated costs of medication, while negative deviations mean that psychiatrists underestimated these costs. In a similar manner, gaps were calculated (in percentages) between the psychiatrists’ estimation of National Insurance Institute disability pension and actual pension received.

The second group of dependent variables included self-reported questions, in which psychiatrists were asked about their attitudes towards various statements regarding costs of psychotropic medications (e.g., level of confidence of their subjective estimations).

To assess differences between participants who worked in various regions, the districts Haifa, North, Jerusalem and South were grouped into a category we called “Other”, and Tel Aviv and Center were merged to a category we called “Center”. This grouping was conducted owing to the discrepancies in income, costs of living and availability of medical care in the peripheral districts as compared to Tel-Aviv and its environs. Individuals who do not live in the center tend to have lower income and often have less availability of local medical care. A larger portion of the population in the periphery reported that they skipped medical treatment for financial reason compared to populations in the center of Israel (19% vs. 6%, respectively). In addition, more households are at risk for poverty in the periphery (37% vs. 22%, respectively) [11].

To assess differences between two groups (e.g., sex) in both dependent variable groups, Mann–Whitney tests were conducted. Psychiatrists who underwent combined specialization were excluded from the analyses because of the small numbers. To assess differences between three groups or above (e.g., work-places) in both dependent variable groups, Kruskal–Wallis tests were conducted. Associations between two categorical variables were assessed using Chi-square tests. Finally, Pearson correlations were calculated between the study variables. The level of significance for all analyses was 5%. Data were analyzed using the Statistical Package for the Social Sciences (SPSS) Statistics for Windows, Version 25.0. (Armonk, NY, IBM Corp., released 2017).

## Results

Table 1 shows demographic characteristics of psychiatrists in the sample (N = 175). The majority were men (55%), born in Israel (73.7%), and a similar rate of physicians had studied medicine in Israel. Of those working in the public sector, the majority worked in hospitals (n = 57, 57%). Most of the sample were adult psychiatrists, including those who did integrated specialization of both adult and child and adolescent psychiatry (n = 95, 81.5%).

**Table 1:** Demographic characteristics of study sample (N = 175)

Variable	N	%	Mean	Standard deviation	Missing
Sex					38
Male	76	55			
Female	61	44			
Age			47	11.89	45
Country of birth					43
Israel	97	73.7			
Abroad	35	26.5			
Marital status					38
Single	14	10.2			
Married	110	80.3			
Divorce	12	8.8			
Widow	1	< 1			
Children			2.63	1.32	61
Study institute location					43
Israel	97	73.5			
Abroad	35	26.5			
Residency institute location					61
Israel	112	98.2			
Abroad	2	1.8			
Specialization type					58
Adult psychiatry	87	74.7			
Child & adolescent psychiatry	22	18.8			
Integrated specialization (adults + child and adolescent)	8	6.8			
Level of specialization					58
Resident	24	20.5			
Specialized	93	79.5			
Residency’s stage					–
Before stage A exam	10	41.7			
Before final exam	7	29.2			
After final exam	7	29.2			
District					78
North	7	7.2			
Haifa	12	12.4			
Tel Aviv	20	20			
Jerusalem	9	9.3			
Center	40	41.2			
South	9	9.3			
Distribution of public service work					
Hospital	57	57			75
Community clinic	23	23			
Hospital clinic	20	20			

### Gaps between subjective estimations and objective costs of psychiatric treatment

Table 2 shows the level of knowledge of psychiatrists regarding patients’ National Insurance Institute disability pension, HMO coverage and the patient’s deductible. In addition, the table presents physicians' estimates of the costs of selected psychiatric drugs.

**Table 2:** Gaps between subjective and objective mental health treatment costs

Service/treatment	Exact value in NIS	Subjective Estimation (NIS)			Gap (%)			Over/under estimation
		M	SD	Range	M	SD	Range	
National Insurance Disability Pension	2350	2887.08	622.97	1400–5400	22.85	26.51	− 40.43–129.79	Over
Maximum HMO coverage of medication expenses	334.5	429.61	288.57	20–1000	28.43	86.27	− 94.02–198.95	Over
Medications (milligram)								
Olanzapine/Zyprexa (5)	87.14	122.5	104.24	17–500	40.58	119.63	− 80.49–473.79	Over
Olanzapine/Zyprexa (10)	142.19	157.59	115.99	17–500	10.83	81.57	− 88.04–251.64	Over
Risperidone/Risperidex (1)	28.9	56.65	36.44	10–150	96.03	126.10	− 65.40–419.04	Over
Paliperidone/Xeplion (100)	1852.79	491.82	369.09	17–1600	− 73.45	19.92	− 99.08–13.64	Under
Perphenan (8)	32.14	25.89	12.91	2–60	− 20.14	39.81	− 93.83–85.07	Under
Ariply (10)	247.1	143.42	111.04	17–500	− 41. 96	44.94	− 93.12–102.35	Under
Mean anti-psychotics	–	–	–	–	6.50	57.14	− 79.56–163.53	Over
Venlafaxine/Venla XR (75)	33.69	75.48	50.95	7–200	124.03	151.22	− 79.22–493.55	Over
Escitalopram/Cipralex (10)	37.42	57.82	33.25	10–150	54.53	88.85	− 73.28–300.86	Over
Bupropion/Wellbutrin XR (150)	106	112.5	61.12	10–250	6.14	57.66	− 90.57–135.85	Over
Mean Anti-depressants	–	–	–	–	67.08	91.37	− 81.02–397.25	Over
Ritalin (10)	30.1	50.36	32.1	10–150	67.29	106.65	− 66.78–398.34	Over
Ritalin LA (20)	158.36	144.43	93.6	20–400	− 8.8	59.1	− 87.37–152.59	Under
Concerta (54)	281.41	216.34	123.18	20–500	− 23.12	43.77	− 92.89–77.68	Under
Mean amphetamines	–	–	–	–	12.34	54.00	− 78.36–164.93	Over
Cannabis (20)	370	275.15	136.89	30–600	− 25.63	37	− 91.89–62.16	Under
Personal participation								
Average deductible and HMO coverage ratio	85	57.81	26.51	50–100	− 31.99	31.18	− 99.41–17.65	Under

As shown in Table 2, there was generally a large overestimation of both National Insurance Institute disability pension (M = 22.85%) and maximum HMO coverage (M = 28.43%), with larger heterogeneity among participants regarding HMO coverage.

As for medications, while some drugs were overestimated (e.g., Olanzapine/Zyprexa), others were underestimated (e.g., Paliperidone/Xeplion). All anti-depressant medications were overestimated, some substantially (e.g., average deviation for Venlafaxine/VenlaXR 75 mg was 124.03%). Regarding ADHD medication- amphetamine stimulants, the price of the two more expensive medications (Methylphenidate/Ritalin LA 20 mg and Methylphenidate HCl ER/Concerta 54 mg) was underestimated.

As a group, the highest estimation gap was found for antidepressants (67.08%, SD 91.37) and the lowest for antipsychotics (6.5%, SD 7.14).

### Associations between demographic variables and treatment estimations

In general, there was no consistent gender difference regarding treatment cost estimations.

Israeli born psychiatrists overestimated National Insurance Institute disability pension when compared to psychiatrists born abroad (Mean gap 26.13, SD = 23.8 vs. 16.3, SD = 33.5, *p* = 0.04). Psychiatrists who studied in Israel overestimated *maximum* HMO coverage (35.66, SD = 85.3 vs. 13.21, SD = 94.4, *p* = 0.045) and underestimated the *average* HMO coverage ratio in comparison to psychiatrists who studied abroad (− 35.9, SD = 30.6 vs. − 18.21, SD = 29.6, *p* = 0.01). No other differences were found in estimations of medication costs regarding these two comparisons.

Compared to child and adolescent psychiatrists, adult psychiatrists overestimated National Insurance Institute disability pension (25.35 vs.11.61, *p* = 0.02), underestimated anti-depressant price (54.45 vs. 105.12, *p* = 0.01) and marginally significantly underestimated anti-psychotic prices (0.30 vs. 21.1299 *p* = 0.055). No other significant differences were found in estimations of medical treatments. Psychiatrists who worked in hospitals overestimated the price of anti-depressants and anti-psychotics in comparison to psychiatrists who worked in clinics, both community and hospital clinics (69.2, SD = 82.7 vs. 66.5, SD = 54.5, *p* = 0.01, 9.75, SD = 52.9, vs. 1.27, *p* = 0.055, respectively).

Regarding work area, the only significant difference was that psychiatrists who did not work in the center underestimated the price of cannabis (− 33.25, SD = 35.8 vs. − 14.03, SD = 38.7, *p* = 0.02) when compared to centrally located physicians.

### Attitudes towards treatment costs

As shown in Table 3, average level of confidence of psychiatrists in their cost estimations was moderate (2.25, SD = 0.95, on 1–5 scale), while they rated the importance of this knowledge as high (4.45, SD = 0.73, on 1–5 scale). Psychiatrists attributed relatively similar weights to price consideration (3.89), patient ability to purchase medications (3.67) and ease of access to information (3.20). Regarding attitudes towards treatment costs according to region (center vs. other), psychiatrists who did not work in the center were more considerate of the cost of the drug (M other = 4.23, SD = 0.8, M center = 3.77, SD = 0.96), and more considerate of the patient's ability to buy medication (M other = 4.05, SD = 0.88, M center = 3.48, SD = 0.96). Other socio-demographic characteristics were unrelated to those attitudes.

**Table 3:** Descriptive statistics of self-report measures

	M	SD	1—Lowest	2	3	4	5—Highest	**%**	**N**
Confidence level in medications assessments	2.3	0.95	33 (26.40%)	39 (31.20%)	42 (33.60%)	11 (8.80%)	0		
Level of importance of knowing the price of medication	4.5	0.73	1 (0.80%)	2 (1.60%)	6 (4.80%)	47 (37.60%)	69 (55.20%)		
Price consideration	3.9	0.97	1 (0.80%)	11 (8.90%)	27 (21.80%)	47 (37.90%)	38 (30.60%)		
Patient ability to purchase medications	3.7	0.99	3 (2.40%)	12 (9.60%)	29 (23.20%)	60 (48.005)	21 (16.80%)		
Ease of access to information	3.2	1.08	8 (6.50%)	23 (18.50%)	44 (35.50%)	34 (27.40%)	15 (12.10%)		
Source of information—patients								77.4	96
Source of information—pharma companies								56.5	70
Source of information—HMO funds								33.9	42
Source of information—pharmaceutical networks								29	36
Source of information—other								10.5	13

The two most common sources of information for medication costs were patients (77.4%) and pharmaceutical companies (56.5%). Regarding information source, a significant difference was detected for two demographic characteristics: Considerably more psychiatrists who were born in Israel and who had studied in Israel used websites, compared to their counterparts (36.9% vs 10.0% and 36.6% vs. 14.3%, relatively).

### Associations between study variables

As shown in Table 4, a positive Pearson correlation was found between National Insurance Institute disability pension gap and mean medications gap (r = 0.25, *p* < 0.01), i.e., large overestimation of National Insurance Institute disability pension was associated with large overestimation of the cost of medication. In addition, ease of access to information was negatively related to mean medications gap (r = − 0.18, *p* < 0.05), as well as maximum coverage by HMO gap (r = − 0.29, *p* < 0.05) and average HMO coverage ratio gap (r = − 0.28, *p* < 0.05). There was a positive and significant correlation between the level of confidence in knowledge of medication costs and the degree of importance of the cost of medications (r = 0.24, *p* < 0.05), and considerations of cost when prescribing therapy.

**Table 4:** Pearson correlations between study variables

	1	2	3	4	5	6	7	8
1. National Insurance Disability gap								
2. Maximum participation of HMO funds gap	0.13							
3. Mean medications gap	0.25**	−0 .13						
4. Average participation HMO ratio gap	0.02	−0 .20	0.13					
5. Confidence level in medications assessments	0.15	0.05	0.08	0.66				
6. Level of importance of knowing the price of medication	0.08	0.13	−0 .13	−0.92	0.24**			
7. Price consideration	0.05	0.03	− 0.90	−0.21*	0.39**	0.34**		
8. Patient’s ability to purchase medications	−0 .04	−0.08	− 0.60	0.53	0.33**	0.14	0.48**	
9. Ease of access to information	0.08	−0 .29*	−0.18*	−0.28*	0.004	0.09	0.20**	0.03

^*^p < .05, **p < .01

## Discussion

In the current study, the overall knowledge of psychiatrists regarding financial resources and health care expenses of their patients was low, as reflected by significant heterogeneity regarding cost of medications (both over and under-estimation of cost) and overestimation of both disability pension and HMO coverage. Psychiatrists were aware of the gaps in their level of knowledge regarding the costs of medications and the importance of this knowledge in prescribing these medications. The low overall knowledge of psychiatrists regarding the cost of medication is in line with previous studies, regarding physicians in general and psychiatrists in particular [5, 8]. The combination of lack of knowledge of both expenses (medication costs and limitations of HMO coverage) and income (National Insurance Institute disability pension) may lead to non-affordable treatment plans, despite the facts that healthcare in Israel is universal, and HMO medical insurance plans include mental health services and some psychotropic medications. This is especially true if the expenditure is out-of-pocket, and not covered by the National Basket committee or by HMO (e.g., when prescribing a new medication not yet included in the basket of approved medications, or when prescribing a medication for an indication other than that approved by the HMO). In this context, it is important to note that patients’ concerns about cost of medications is one of the reasons for non-adherence [12]. This may result in partial or total non-compliance of patients with medical recommendations, based on inability to afford the cost of treatment. Inability to afford treatment may also limit access to mental health services (e.g., patients may be too embarrassed to keep appointments that they cannot afford, or may not share the information that they did not purchase medication). Non-adherence in turn may promote deterioration of clinical and economic outcomes (e.g., chronicity, disability, lack of remission, need for hospitalization instead of community treatment, loss of work days). On a national scale, non-adherence to treatment may impact the economic burden and economic growth, including increased direct and indirect costs related to mental illness (e.g., unemployment, lack of productivity) [13].

In demographic comparisons, psychiatrists who did not work in the center (but rather in other areas) paid more attention to medication costs, compared to those who worked in the center of Israel. This may indicate that doctors whose patients are of lower socio-economic status need to be more sensitive to economic issues in treatment planning. A better knowledge of expenses and financial resources by child psychiatrists may reflect habitual cooperation with social services.

Most psychiatrists in our sample reported that they based their knowledge regarding medication costs on their patients’ reports (77%) or from pharmaceutical companies (57%). This vast reliance on patients’ reports and pharmaceutical companies for medication prices may limit knowledge of the true cost of medications, or generate biased concepts. The ease of access to information as reported by psychiatrists was significantly negatively associated with the mean medications gap. This means that psychiatrists who reported easier access to information regarding medication costs were found to have smaller gaps between their assumed and actual costs. The more confident psychiatrists were in their knowledge of treatment costs, the more importance they attributed to this issue, and the more sensitive they were to cost considerations when prescribing pharmacotherapy. These findings support the great importance of improving the accessibility of medication costs to psychiatrists.

This study has several limitations. We could not measure the response rate to our questionnaires, nor to characterize and compare those who responded and those who did not (selection bias). This is due to unavailable information (exact number of psychiatrists – specialists and residents) and the study methodology (i.e., email recruitment, anonymity). It is possible that some psychiatrists did not receive the study questionnaire. To increase response rates, answering all questions of the questionnaire was not mandatory. Therefore, for some questions remained unanswered, and there were missing answers. In any case, the response rate was low, and may not truly represent Israeli psychiatrists (a threat to external validity). However, it is possible that this non-representative sample may indicate that the true knowledge of psychiatrists regarding treatment costs may be even lower, as those who chose to participate (and were aware of the study’s main questions) are more aware of the importance of these issues. This current analytical cross-sectional study, did not enable us to determine causality. However, we believe the study’s results reflect general associations and trends.

Although most psychiatrists reported that considering the cost of medication is important or highly important, we did not examine other considerations in the process of drug prescription (e.g., clinical indications, efficacy). This could represent a social-desirability bias.

## Conclusions

To conclude, this is the first national survey in Israel to examine psychiatrists’ levels of knowledge of drug costs and disability pension, and their assessment of the impact of these factors on their treatment decisions. In terms of policy, in light of the insufficient knowledge revealed, psychiatrists should be better informed of economic issues associated with patient care, cost of pharmacological treatments and disability pensions. These topics should be included in training programs beginning with medical schools, psychiatry residency and continuous education programs. Government ministries (health, welfare), HMOs and other health care providers should be encouraged to allow easily accessible sources of cost information for psychiatrists, patients and their families. Directors of mental health clinics and psychiatric departments should be encouraged to regularly provide updated treatment related economic information in their workplaces. Further research is needed to ensure implementation of health-care related economic information.

## Data Availability

Data and questionnaires are available from the corresponding author upon request.

## Abbreviations

ADHD: Attention deficit hyperactivity disorder
GP: General practitioner
HMO: Health maintenance organizations
IMA: Israel Medical Association
IPA: Israel Psychiatric Association
IRB: Institutional review board
SPSS: Statistical Package for the Social Sciences
US: United States

## Footnotes

1 https://www.health.gov.il/Subjects/Finance/DrugPrice/Pages/default.aspx [Hebrew].
